# 15q13.3 homozygous knockout mouse model display epilepsy-, autism- and schizophrenia-related phenotypes

**DOI:** 10.1038/tp.2016.125

**Published:** 2016-07-26

**Authors:** A Forsingdal, K Fejgin, V Nielsen, T Werge, J Nielsen

**Affiliations:** 1 Synaptic Transmission, In Vitro, Neuroscience Research DK, H. Lundbeck A/S, Valby, Denmark; 2 Institute of Biological Psychiatry, Mental Health Center, Sct. Hans, Mental Health Services, Roskilde, Denmark; 3 Institute of Clinical Sciences, Faculty of Medicine and Health Sciences, University of Copenhagen, Copenhagen, Denmark; 4 iPSYCH, The Lundbeck Foundation’s Initiative for Integrative Psychiatric Research, Denmark

## Abstract

The 15q13.3 microdeletion syndrome is caused by a 1.5-MB hemizygous microdeletion located on 15q13.3 affecting seven genes: *FAN1*; *MTMR10*; *TRPM1*; *miR-211*; *KLF13*; *OTUD7A*; and *CHRNA7*. The 15q13.3 microdeletion increases the risk of intellectual disability, epilepsy, autism spectrum disorder and schizophrenia, though the clinical profile varies considerably. Two mouse models of this syndrome, with hemizygous deletion of the orthologous region in the murine genome, have recently been shown to recapitulate a number of the behavioral and physiological deficits that characterize the human condition. Still, little is known of the underlying biological mechanisms. Eleven human cases with homozygous deletion of the 15q13.3 region have been reported, all with severe functional and physiological impairments. We therefore hypothesized that a 15q13.3 homozygous knockout would confer more pronounced behavioral and physiological deficits in mice than the 15q13.3 hemizygous deletion. Here we report the characterization of a 15q13.3 knockout mouse. We observed marked deficits including altered seizure susceptibility, autistic behavior-related phenotypes, and auditory sensory processing. Several of these deficits, albeit less pronounced, were also found in the 15q13.3 hemizygous littermates indicating a gene-dosage dependency. Our findings strongly indicate that studies of the hemi- and homozygous 15q13.3 mouse strains will facilitate understanding of the biological mechanisms of severe mental disorders.

## Introduction

Decades of intensive research have provided little knowledge and understanding of the biological changes underlying severe psychiatric disorders, in particular schizophrenia and autism. One advancement has come from genome-wide association studies that identified hemizygous microdeletions and microduplications termed copy-number variants (CNVs), which confer high risk of these disorders.^1^ The 15q13.3 hemizygous microdeletion increases the risk of intellectual disability, epilepsy, autism spectrum disorder and schizophrenia 10-fold or more.^2, 3, 4, 5^ The microdeletion encompasses a region of ~1.5 MB from break-point (BP) 4 to BP5, with seven genes; *MTMR10*; *FAN1*; *TRPM1*; *miR-211*; *KLF13*; *OTUD7A*; and *CHRNA7* (OMIM #612001). The clinical phenotype varies greatly among individuals with the 15q13.3 microdeletion, ranging from asymptomatic to severe neuropsychiatric disorders. In addition, the microdeletion is present in ~1% of patients with idiopathic generalized epilepsy compared with 0.019% in the general population.^1, 4, 6^ Epileptic carriers that have been phenotyped in detail mainly suffer from absence seizures and not from generalized seizures.^7, 8^

Disorder-associated CNVs provide an opportunity to gain insight into underlying mechanisms of the pathogenesis. We, and others, recently generated and characterized mouse models of the 15q13.3 microdeletion syndrome by deleting the orthologous genomic region on mouse chromosome 7.^9, 10^ Behavioral characterization of the 15q13.3 mouse models, Df(h15q13)^+/−^, revealed schizophrenia-, autism- and epilepsy-related phenotypes.^9, 10^

The 15q13.3 CNV is distinct from most known high-risk CNVs as relatively few genes are involved, simplifying mechanistic evaluation. Furthermore, humans with homozygous deletions are viable, as 11 cases of homozygous deletion of the 15q13.3 region have been reported, all of them with severe physical and mental handicaps.^11^

The viability of humans with homozygous 15q13.3 deletion suggests that the generation of a homozygous mouse model for this syndrome is feasible. We hypothesize that a 15q13.3 homozygous mouse model will provide both new and stronger phenotypes than those previously observed in the hemizygous mouse models, and that the combined study of wildtype (WT), hemizygous and homozygous 15q13.3 mouse strains will inform on the molecular and physiological mechanisms underlying schizophrenia, autism and epilepsy.

Here we present, to the best of our knowledge, the first study of homozygous 15q13.3 knockout mice, Df(h15q13)^−/−^ mice, in which we compare Df(h15q13)^−/−^, Df(h15q13)^+/−^ and WT littermates. We focus on behavioral assays related to the neuropsychiatric disorders known to affect human 15q13.3 microdeletion carriers.

## Materials and methods

### Animals

Df(h15q13)^−/−^ mice were bred by mating Df(h15q13)^+/−^ mice. The generation of Df(h15q13)^+/−^ mice was described by Fejgin *et al.*^9^ At weaning, when mice were 3 or 4 weeks of age, biopsies were collected for PCR-based genotyping (see next paragraph). Mice were then split by gender and group housed (mixed genotypes from the same litter, up to seven animals per cage), except during the Nest-building assay where animals were single housed. Mice were bred, genotyped and housed until the age of 8 weeks at Taconic MB (Lille Skensved, Denmark), except for the pups used for ultrasonic vocalization recordings. As the genotype assigned animals to groups, randomization of animals to experimental groups was not relevant. At 8 weeks of age, the mice were transferred to the Lundbeck facility. Animals were housed in Macrolon (type II) cages with standard sawdust bedding and environmental enrichment (plastic igloo and wooden chew blocks) in a 12-h light cycle starting at 0600 hours and a temperature of 21±2 °C; and humidity of 55±5%. Food (Altromin 1323 pills, Brogaarden, Denmark) and tap water were available *ad libitum*. Animals were allowed to acclimatize to the facility for at least 5 days before any test. Experimenters were blinded to the genotype. Testing was conducted using male and female mice between 10 and 22 weeks of age unless otherwise stated. All the data shown is from males unless otherwise stated. Sample sizes for each experiment can be seen in Table 1. Sample sizes were generally determined by how many homozygous mice could be bred (and survived). All studies were carried out in accordance with Danish legislation, granted by the animal welfare committee, appointed by the Danish Ministry of Food, Agriculture and Fisheries—Danish Veterinary and Food Administration.

### Genotyping and confirmation of gene deletion

PCR was carried out as described in Fejgin *et al.*^9^ Tissue was isolated from whole brain. Total RNA was purified using the NucleoSpin RNA and Protein kit according to manufacturer’s instructions (Macherey-Nagel, Düren, Germany) followed by first strand complementary DNA synthesis using TaqMan reverse transcription reagents according to manufacturer (Life Technologies, Paisley, UK). Primers designed and validated by Fejgin *et al.*^9^ were used. Quantitative PCR was performed on a BioRad C1000 Touch thermal cycler with a CFX384 optical reaction module using SsoFast EvaGreen Supermix according to manufacturer (BioRad, Hercules, CA, USA). Eight reference genes were included of which three were selected (*Actb*, *Gapdh* and *H3f3a*) for geometric normalization according to geNorm software version 3.5.^12^ Transcripts with Cq values >35 were considered below the detection limit and removed. Genes close to the deleted region were included for detection of possible effects on bordering genes. The number of genes upstream and downstream of the deleted region was increased until transcripts could be detected.

### Irwin test

Basic behavioral functions and neurological reflexes, such as vision, fear responses, muscle coordination and so on were tested by a simplified version of the original screening described by Irwin.^13^ We measured: (1) undisturbed behavioral observation in a small observation cage (following 10-min habituation to the cage)—body position, bizarre behavior (compulsive/self-bite, circling and head movements) and tremors. (2) Response to finger approach (withdrawal and explorative responses to a slow finger approach). (3) Touch escape (response to the gentle pressure over the sides and back of the mouse). (4) Grip strength (resistance of the animal to pull when holding on a wire-mesh grid). (5) Visual placing response (animal is lifted vertically by mid-tail about 15 cm above the wire-mesh grid and lowered slowly to elicit limb extension). (6) Corneal response (response to the slow approach of a toothpick to the eye, on both sites). (7) Toe-pinch response (elicited by light compression of the lateral surface of the mid-digit of each foot with a forceps). (8) Wire-maneuver (animal is brought to the horizontal wire, allowed to grasp it with its forelimbs, then released and observed). (9) Limb and abdominal tone (detected by palpation). (10) Tail-pinch response (response to a moderate pressure applied with a forceps, ~2.5 cm below the base of the tail).

### Grip strength

Grip strength was measured using a grip strength meter and peak amplifier (47105-001 Ugo Basile, Varese, Italy) with a metal wire. Mice were held by the tail and lowered toward the wire to allow grasping by the forelimbs. With the body perpendicular to the wire, the mouse was pulled steadily backwards until the grip was released, and the maximal grip strength was recorded.

### Open field

Individual mice were placed in a circular open-field arena (74 cm diameter) for 1 h with low illumination (14 lux). Movement was automatically video tracked and quantified using the Ethovision 7.0 software (Noldus Information Technologies, Wageningen, The Netherlands).

### PTZ-induced seizure

To assess seizure susceptibility in the Df(h15q13)^−/−^ mice we scored 4 seizure behaviors after administration of the convulsant drug pentylene tetrazole (PTZ); (1) early-stage seizures, (2) myoclonic jerks, (3) clonic seizures and (4) clonic–tonic seizures.^9^ After the injection of Pentylene tetrazol (PTZ) (Sigma-Aldrich, Brøndby, Denmark; 40 mg kg^−1^, subcutaneously), each mouse was observed and video recorded for 30 min by an observer blinded to genotype. Video recordings were scored using the Observer software (Noldus Information Technologies). Early-stage seizure was scored as the first four stages of the Racine scale: (1) mouth and facial movements; (2) head nodding; (3) forelimb clonus; (4) rearing.^14^

### Behavior quantification

Animals were placed in separate cages and video recorded for 10 min. Grooming, digging, rearing, jumping/crawling in the corner of the cage and crawling in the lid was manually scored and quantified off-line using the Observer software (Noldus Information Technologies).

### Three-chambered social approach task

The three-chambered social approach task was carried out following the protocol presented by Yang *et al.*^15^ In this test, the mice can move freely between three chambers; an empty chamber in the middle, a chamber with an empty cylinder on one side and a chamber with a novel mouse in a cylinder in the other side. Briefly, the mice were first habituated to the center chamber for 10 min, second, they were habituated to all three chambers for 10 min. Finally, an empty cylinder was put on one of the side chambers and a cylinder with a novel mouse was put on the other side chamber, after which the test for social sniffing was carried out for 10 min.

### Ultrasonic vocalization

Ultrasonic vocalization was recorded as described by Scattoni *et al.*^16^ Pregnant dams were shipped from Taconic to the Lundbeck facility 2 weeks after conception. At postnatal day 6, the pups were put into a sound-isolated box one by one where vocalization was recorded for 3 min with an ultrasound microphone (CM16/CMPA, Avisoft Bioacoustics, Glienicke, Germany) and preamplifier (116H, Avisoft Bioacoustics). After recording, the pup was marked and put back to the dam and littermates. When all pups had been recorded, tail biopsies were obtained and genotyped by PCR. Ultrasonic calls were quantified using the Avisoft-SASLab Pro software (Avisoft Bioacoustics).

### Nest building

Mice were habituated for 2 weeks without nesting material in the home cages. On the day of testing, mice were divided into single cages with an igloo with three openings in one corner and a single square of nesting material (Nestlets, Ancare, Belmont, NY, USA) in the opposite corner of the cage. Nest building was scored every hour during the first 12 h of testing and after 24 h, using the ‘Nesting Index Score’, (NIS), introduced by Pedersen *et al.*^17^ NIS score has two components: (1) how much nest-building material is used (0–5 points); (2) how many openings of the igloo are covered (0–3). Maximal score is 8.

### Progressive acoustic startle

Progressive acoustic startle was tested using the SM100 Startle Monitor System (Kinder Scientific, Poway, CA, USA), consisting of eight sound-attenuated startle chambers and StartleMonitor software (Kinder Scientific). Animals were placed in an adjustable Plexiglas holder, providing limited movement but not restraint, positioned directly above a sensing platform registering the animals startle response. Each test session consisted of a 5-min acclimatization period with only background white noise (62 dB), followed by startle pulses at intensities varying from 95 to 120 dB, each presented 12 times in a balanced manner. Intertrial intervals (ITIs) varied between 9 and 21 s (average ITI 15 s). The full acoustic startle test lasted ~25 min.

### Prepulse inhibition

Prepulse inhibition (PPI) was tested using the same equipment as for the progressive acoustic startle test and as described by Fejgin *et al.*^9^ Each test session consisted of a 5-min acclimatization period with only background white noise (62 dB), followed by a brief habituation setting where 32 regularly occurring startle pulses of 105 dB (ITI: 10 s) were presented to the animals to maximize habituation prior to the PPI part of the session. Animals were then subjected to 5 types of trials presented 12 times each in a balanced manner: pulse alone; prepulse+pulse (5, 10 or 15 dB above background); or highest prepulse intensity (77 dB) alone. ITI varied between 9 and 21 s (average ITI 15 s) and interstimulus interval was set to 100 ms with a prepulse length of 20 ms. Each PPI session ended with eight startle pulses of 105 dB to estimate habituation across PPI trials. The full PPI test lasted about 28 min. PPI was calculated as % PPI for each prepulse intensity as: 100−((prepulse+pulse/pulse alone) × 100), that is, a lower percentage score indicates a decrease in PPI. Startle magnitude was calculated as an average of pulse alone trials.

### Morris water maze

The apparatus has been described earlier.^18^ One week prior to water maze testing the animals were habituated for 1 min (20 s in 1-cm water and 40 s of handling) for 5 consecutive days. Animals were subjected to four trials per day for 6 consecutive days, with ITI of 30 min. Before the first trial on the first day, the animals were placed on the platform for 10 s. The first trial started immediately thereafter. During any trial, if the mice did not find the platform they were guided to it and left there for 5 s before being lifted out of the maze. For each trial, animals started from randomized positions and were allowed a maximum time of 60 s to find the hidden platform placed in the middle of the northern quadrant.

On the seventh day, a 60-s probe test was performed with the platform removed, followed by a visual test where the platform was marked with a visible flag. After each trial, mice were dried with paper towels and allowed to heat under a heating lamp for 3–4 min before resting in their home cage. Time spent before finding the platform was analyzed using the Ethovision 3.0 software (Noldus).

### Contextual and cued fear conditioning

Training and contextual testing was conducted in a sound-proof chamber (30 × 20 × 40 cm) connected to a ventilation system in an isolated room. The floor of the chamber consisted of a metal grid attached to an electric shock generator. Prior to training and testing, the chamber was cleaned with 70% ethanol. A video camera was used for recording of sessions for subsequent off-line analysis. The test program (shock, sound and light) was run by FCONwin (Ellegaard Systems, Faaborg, Denmark). The training session lasted 6 min. During the first and last minute of the training there was only white light. After 1 min and 40 s, a tone was presented for 20 s (80 db, 2000 Hz). During the last 2 s of the tone the mice received a shock through the grid floor (0.6 mA). The context testing was performed 24 h later. The animals were placed in the same box as previously. The white light was on and the animal was video recorded for 3 min. The cue test was performed 4 h after the context test. The animals were placed in a sound-proof chamber similar to the training and context chamber, but with a white inner box instead of the metal one with grid floor. When an animal was inside the box, the red light was turned on. The animal was left in the box for 1 min before the tone was initiated. The tone (80 dB, 2000 Hz) was on for 2 min after which the animal was left in the box for another minute. Freezing during training, context test and cue test was scored manually using the Observer software (Noldus Information Technologies).

### Data and statistical analysis

Statistical tests are indicated in the respective figure legends. All tests were carried out in Graphpad Prism (La Jolla, CA, USA) and excel, and *P*<0.05 was considered statistically significant. The three genotypes were generally compared by one-way or two-way analysis of variances, if the variance did not differ between the three genotype groups. If the variance did differ between the three groups, they were compared by the non-parametric Kruskal–Wallis test. If overall comparison of genotype groups was significant multiple comparisons were computed. Data shown are from males, unless otherwise stated.

## Results

### Decreased survival and body weight of Df(h15q13)^−/−^ mice

Expression of deleted genes was examined in whole brain by quantitative PCR. All six protein-coding genes in the deleted region were absent in the Df(h15q13)^−/−^ mice, whereas all except Trpm1, which is not normally expressed in the brain, were expressed in WT littermates (data not shown).

**Figure 1:**
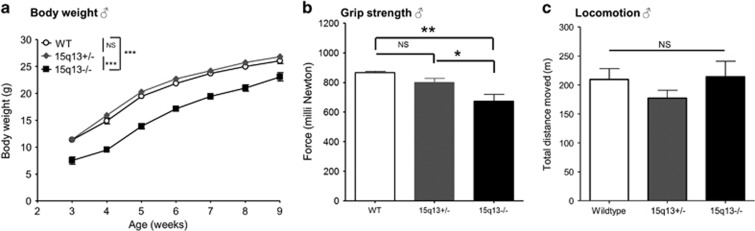
Basic characterization of Df(h15q13)^−/−^ mice. (**a**) Body weight gain. *n*=9–52 for each genotype. Repeated measures ANOVA, *P*<0.0001. Tukey’s multiple comparisons indicated. (**b**) Grip strength measured by Newton meter. *n*=4–6 for each genotype. One-way ANOVA, *P*<0.01. Tukey’s multiple comparisons indicated. (**c**) Locomotion was assessed in an open-field test. *n*=8–11 for each genotype. One-way ANOVA, NS. Data presented as mean±s.e.m. *P<0.05; **P<0.01; ***P<0.001. ANOVA, analysis of variance; NS, not significant; WT, wildtype.

At birth, the fraction of pups that were homozygous for the deletion, Df(h15q13), was 0.20; slightly, but significantly below the expected ratio of 0.25 (binomial test, *P*<0.05). Survival into adulthood (8 weeks of age) was also reduced, but could be improved by later weaning and addition of nutritional supplements (Table 2). We observed decreased body weight persisting into adulthood (Figure 1a), and a small decrease in adult brain weight of Df(h15q13)^−/−^ mice (Supplementary Figure S1).

### Df(h15q13)^−/−^ mice basic behavior

Homozygous Df(h15q13)^−/−^ mice were compared with hemizygous Df(h15q13)^+/−^ and WT littermates in the Irwin test, which assesses basic behavior and physiological function.^13^ During undisturbed observation Df(h15q13)^−/−^ mice had normal body posture and displayed normal behavior (active, exploring, rearing, sniffing, climbing and grooming). However, some Df(h15q13)^−/−^ mice displayed repetitive behavior (jumping or crawling in the corner of the cage). This observation was followed up by a formalized test in which this repetitive behavior was quantified (see later section ‘Df(h15q13)-/- mice exhibit alterations in autism-related tests’). We found that Df(h15q13)^−/−^ mice had decreased grip strength and performed less well in the wire maneuver test, compared with Df(h15q13)^+/−^ and WT littermates (data not shown). Subsequent quantification of grip strength with a Newton meter confirmed that Df(h15q13)^−/−^ mice had decreased grip strength (Figure 1b). Df(h15q13)^−/−^ mice were normal in all other characteristics assessed by the Irwin test; visual placing response, corneal response, limb and abdominal tone, touch escape, and responses to tail-pinch, toe-pinch and finger approach (data not shown).

To investigate motor activity and anxiety of the Df(h15q13)^−/−^ mice, they were tested in the open-field assay. Df(h15q13)^−/−^ mice moved the same distance as their WT littermates during the 1-h trial (Figure 1c). Other parameters such as velocity, time spent in inner, middle or outer zone and so on, did not differ between genotypes either (data not shown).

Several of the following behavioral assays have been conducted in more than one batch of animals and in both male and female mice. Unless otherwise stated, the data shown in the primary figures are from the batch of males with most animals, and the additional data sets are shown in the Supplementary Figures. Table 3 summarizes the behavioral phenotypes observed in the Df(h15q13)^+/−^ mice.

### Df(h15q13)^−/−^ mice show abnormal susceptibility to PTZ-induced seizures

To assess seizure susceptibility in the Df(h15q13)^−/−^ mice we scored four seizure behaviors after pentylene tetrazole (PTZ) administration; (1) early-stage seizures, (2) myoclonic jerks, (3) clonic seizures and (4) clonic–tonic seizures.

### Early-stage seizures

**Figure 2:**
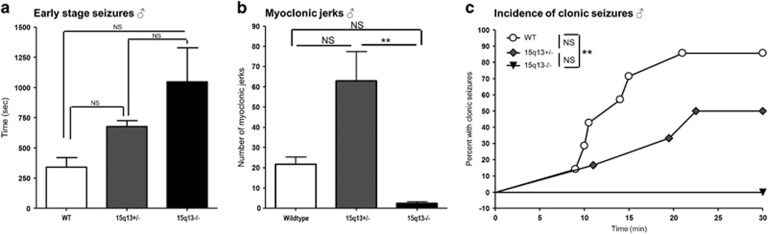
Abnormal seizure response to PTZ in Df(h15q13)^−/−^ mice. Mice received PTZ 40 mg kg^−1^ subcutaneously and were observed for 30 min and scored for seizures as described in Materials and Methods. *n*=5–7 for each genotype. (**a**) Time in early-stage seizure. Kruskal–Wallis test, *P*<0.05. Dunn’s multiple comparisons induicated. (**b**) Number of myoclonic jerks during a 30-min period after PTZ administration. Kruskal–Wallis test, *P*<0.01. Dunn’s multiple comparisons indicated. (**c**) Incidence of clonic seizures. Logrank test, *P*<0.01. WT vs Df(h15q13)^+/−^ NS, WT vs Df(h15q13)^−/−^ *P*<0.01, Df(h15q13)^+/−^ vs Df(h15q13)^−/−^ NS. Data (**a**, **b**) presented as mean±s.e.m. **P<0.01. NS, not significant; PTZ, pentylene tetrazole; WT, wildtype.

The Df(h15q13)^−/−^ mice consistently had at least a twofold increase in time spent in early-stage seizures than their WT littermates. There was also an increase in the time spent in early-stage seizures for the Df(h15q13)^+/−^ mice compared with WT littermates (*t*-test *P*<0.01), although this was not statistically significant when correcting for multiple testing (Figure 2a and Supplementary Figures S2A and D).

To clarify if the genotype difference in time in early-stage seizures was because the WT mice stopped having early-stage seizure behavior after progressing into clonic seizures, we compared time spent in early-stage seizures before any of the animals had developed clonic seizures. During this period the Df(h15q13)^−/−^ mice also spent more time in early-stage seizures (Supplementary Figure S3).

### Myoclonic jerks

As previously reported, the Df(h15q13)^+/−^ mice had significantly increased propensity for myoclonic jerks compared with WT littermates after PTZ administration.^9^ However, the Df(h15q13)^−/−^ mice had few (Figure 2b and Supplementary Figures S2B and E).

### Clonic seizures

We observed increased latency and decreased incidence of clonic seizures induced by PTZ in Df(h15q13)^+/−^ mice, similar to what have previously been reported.^9^ No Df(h15q13)^−/−^ mice had clonic seizures after PTZ administration, indicating that this phenotype is also gene-dosage dependent (Figure 2c and Supplementary Figures S2C and F).

### Clonic–tonic seizures

In both the batches of animals 0–33% of the WT mice with clonic seizures progressed to clonic–tonic seizures, whereas we did not observe clonic–tonic seizures in any of the Df(h15q13)^+/−^ or Df(h15q13)^−/−^ mice (data not shown). There was no difference in PTZ blood levels between genotypes (average=10651 ng ml^−1^, *P*=0.96, measured 1 h after dosing).

We have not observed spontaneous convulsive seizures in home cages or during behavioral tests.

### Df(h15q13)^−/−^ mice exhibit alterations in autism-related tests

**Figure 3:**
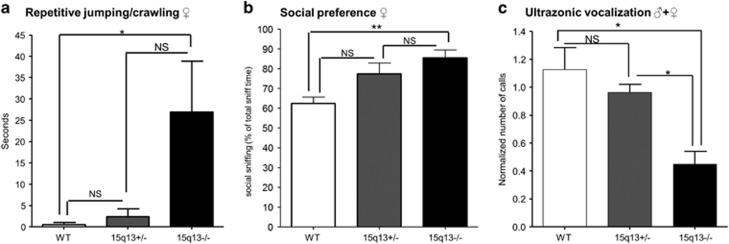
Autistic features in Df(h15q13)^−/−^ mice. (**a**) Mice were placed in individual cages and observed for 10 min. Quantification of repetitive jumping or crawling in the corner of the cage is shown here. *n*=10–13 for each genotype, female mice. Kruskal–Wallis test, *P*<0.05. Dunn’s multiple comparisons indicated. (**b**) Three-chambered social approach task. Social sniffing: time spent sniffing the cylinder with the novel mouse. Total sniffing time: time spent sniffing the cylinder with the novel mouse and the empty cylinder. *n*=6–7 females for each genotype. One-way ANOVA, *P*<0.01. Tukey’s multiple comparisons indicated. (**c**) Ultrasonic vocalization in 6-day-old pups. Calls are normalized to the average number of calls within each litter. *n*=6–53 for each genotype. One-way ANOVA, *P*<0.05. Tukey’s multiple comparisons indicated. Data presented as mean±s.e.m. *P<0.05; **P<0.01. ANOVA, analysis of variance; WT, wildtype.

Stereotypic repetitive movements, in the form of jumping or crawling in the corner of the cage were observed in several homozygotes during the Irwin test and in home cages, primarily in females. Therefore, this behavior was quantified, and Df(h15q13)^−/−^ mice displayed more repetitive crawling or jumping in the corner of the cage than WT and Df(h15q13)^+/−^ littermates (Figure 3a). This was replicated in a separate batch of animals and while the phenotype appeared stronger in female mice a similar pattern was observed in male mice (Supplementary Figure S4). We also quantified other behaviors such as grooming, rearing, digging and so on, but found no difference between the genotypes (data not shown).

In order to address social behavior in the Df(h15q13)^−/−^ mice, we tested them in the three-chambered social approach task.^15^ All animals spent more time sniffing the cylinder with the novel mouse compared with the empty cylinder. Df(h15q13)^−/−^ mice spent 20% more of total sniffing time at the cylinder with the novel mouse than their WT littermates (Figure 3b). There was also a change in the same direction for the Df(h15q13)^+/−^ mice (*t*-test, *P*<0.05), although this was not statistically significant when accounting for multiple testing. There was no difference in total sniffing time (time spent sniffing either of the two cylinders) between genotypes.

We tested ultrasonic vocalization of Df(h15q13)^−/−^ pups, and found that they had a twofold decrease in number of vocalizations Df(h15q13)^+/−^ and WT littermates (Figure 3c). There was also a trend towards a decrease in vocalization for the Df(h15q13)^+/−^ mice, although this was not statistically significant.

### Df(h15q13)^−/−^ mice perform less nest building

**Figure 4:**
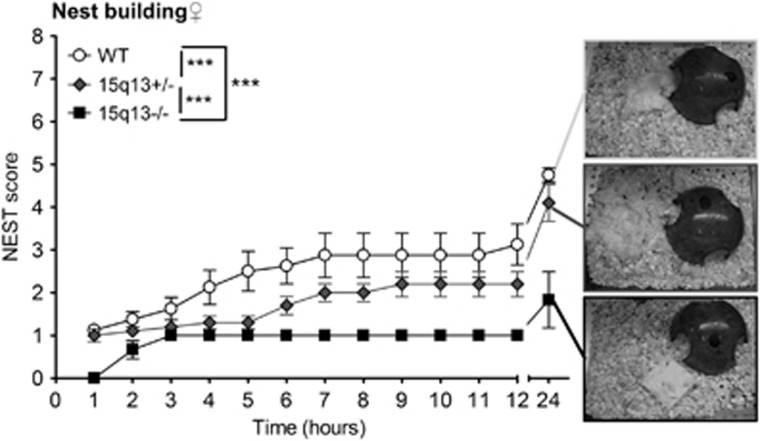
Impaired nest building in Df(h15q13)^−/−^ mice. Maximal score is 8 (see Materials and Methods). *n*=6–10 females for each genotype. Two-way ANOVA, no significant interaction between time and genotype. Main effect of time (*P*<0.0001) and genotype (*P*<0.0001). Tukey’s multiple comparisons indicated. Data presented as mean±s.e.m. ***P<0.001. ANOVA, analysis of variance; WT, wildtype.

We scored nest building in Df(h15q13)^−/−^, Df(h15q13)^+/−^ and WT female mice. In a gene-dosage dependent manner, Df(h15q13) mice performed less nest building than their WT littermates (Figure 4).

### Df(h15q13)^−/−^ mice show alterations in preattentive information processing

We assessed acoustic startle response at varying intensities, and PPI of the acoustic startle response in Df(h15q13)^−/−^ mice compared with Df(h15q13)^+/−^ and WT littermates.

**Figure 5:**
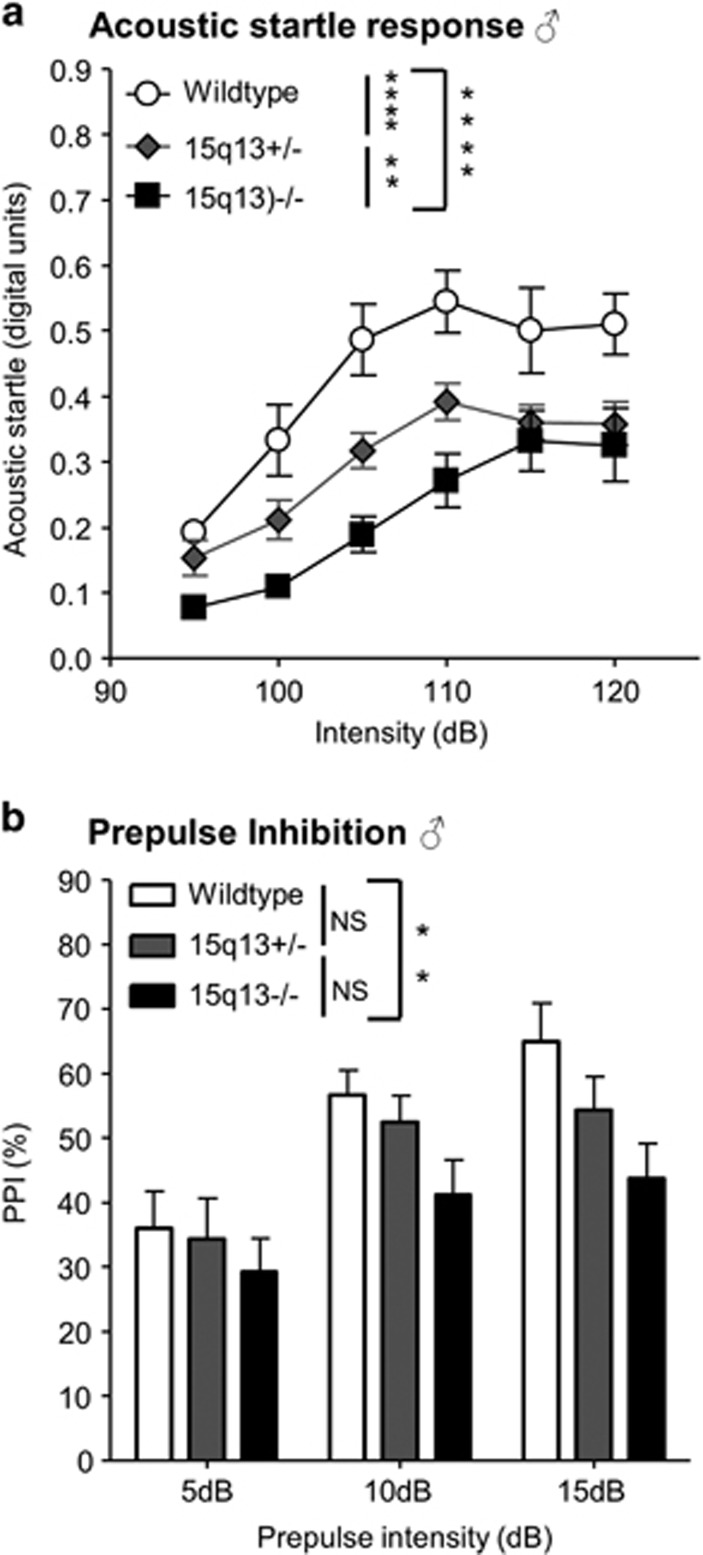
Auditory sensory processing and –gating deficits in Df(h15q13)^−/−^ mice. *n*=8–11 for each genotype. (**a**) Progressive startle. Two-way ANOVA, no significant interaction between intensity and genotype. Main effects of intensity (*P*<0.0001) and genotype (*P*<0.0001). Tukey’s multiple comparisons of genotype groups indicated. (**b**) Prepulse inhibition (PPI) at varying prepulse intensities. Two-way ANOVA, no significant interaction between prepulse intensity and genotype. Main effects of prepulse intensity (*P*<0.0001) and genotype (*P*<0.01). Tukey’s multiple comparisons of genotype groups indicated. **P<0.01; ****P<0.0001. ANOVA, anaysis of variance.

In a gene-dosage-dependent manner Df(h15q13), mice startled less in response to the acoustic stimulus than WT mice (Figure 5a). This pattern was seen in both males and females in two separate batches of animals (Supplementary Figure S5).

Df(h15q13)^−/−^ mice displayed decrease in PPI, that is, impaired sensorimotor gating, compared to WT littermates (Figure 5b). This pattern was also seen in both sexes in two separate batches of animals (Supplementary Figure S6).

### Cognition in Df(h15q13)^−/−^ mice

**Figure 6:**
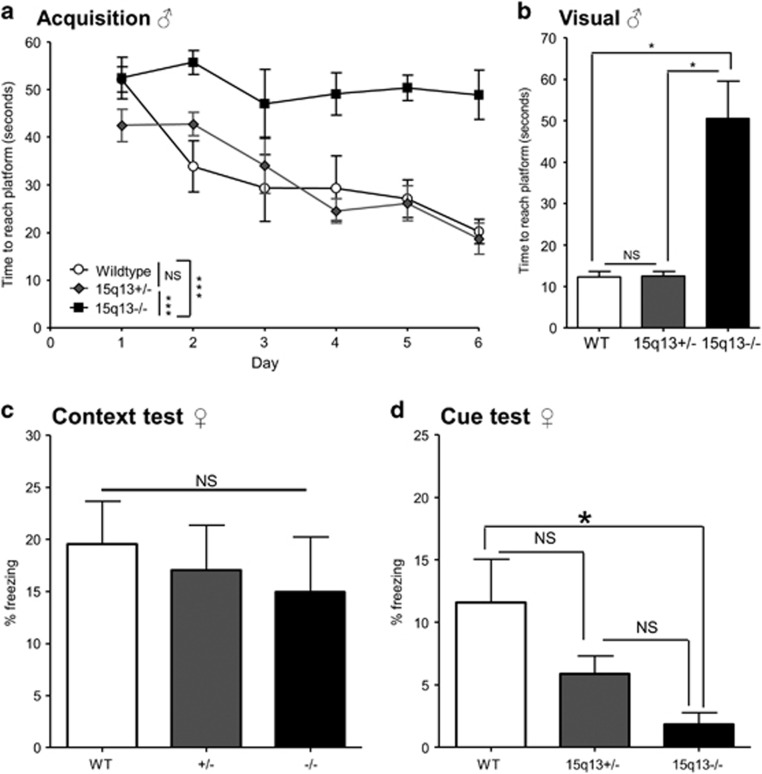
Cognition in Df(h15q13)^−/−^ mice. (**a**, **b**) Morris water maze. *n*=4–6 for each genotype. (**a**) Acquisition for six consecutive days, four trials per day, ITI 30 min. Repeated measures ANOVA, *P*<0.001. Tukey’s multiple comparisons indicated. Mean±s.e.m. (**b**) Visual trial. Kruskal–Wallis test, *P*<0.05. Dunn’s multiple comparisons indicated. (**c**, **d**) Fear conditioning. *N*=6–9 females for each genotype. (**c**) Context fear memory. One-way ANOVA, NS. Mean±s.e.m. (**d**) Auditory cued fear memory. Kruskal–Wallis test, *P*<0.05. Dunn’s multiple comparisons indicated. Data presented as mean±s.e.m. *P<0.05; ***P<0.001. ANOVA, analysis of variance; NS, not significant; WT, wildtype.

To assess spatial memory in the Df(h15q13)^−/−^ mice, we tested them in the Morris water maze and compared their performance with Df(h15q13)^+/−^ and WT littermates. As observed previously, the Df(h15q13)^+/−^ mice were as good as the WT mice at learning where the platform was^9^ (Figure 6a). In contrast, the Df(h15q13)^−/−^ mice did not learn where the platform was (Figure 6a). In the subsequent visual trial, where a flag was placed on top of the platform, the Df(h15q13)^+/−^ mice and WT littermates reached the platform in about 10 s as seen previously,^9^ but the Df(h15q13)^−/−^ mice did not find the platform (Figure 6b). It was not clear whether the inability of the Df(h15q13)^−/−^ mice to learn the location of the platform was due to cognitive and/or visual impairment.

Therefore, we chose to perform a memory test that does not rely strongly on vision—the auditory fear-conditioning test. Contextual fear memory did not differ significantly between the genotypes (Figure 6c). In contrast, auditory cued fear memory was significantly decreased in Df(h15q13)^−/−^ mice compared with WT littermates. There was a change in the same direction for the Df(h15q13)^+/−^ mice, although this was not significant (Figure 6d).

## Discussion

We have shown that Df(h15q13)^−/−^ mice display strong phenotypes related to epilepsy, autism and schizophrenia. Several of these phenotypes were also present in the Df(h15q13)^+/−^ mice, although weaker, indicating a gene-dosage dependency.

Df(h15q13)^−/−^ mice exhibited a strong but complex seizure threshold phenotype in response to the GABA(A) antagonist PTZ with increased propensity for early-stage seizures and a decreased propensity for full clonic seizures. We also found similar, but weaker effects in mice with hemizygous deletion in accordance with earlier work.^9^ In humans, there is also an apparent gene-dosage effect on seizure phenotypes, as humans with hemizygous 15q13.3 deletion have a highly increased risk of epilepsy, while all 11 humans with homozygous deletion developed epilepsy in early childhood. In this context, it may seem unexpected that spontaneous clonic seizures were not observed in the mice, which instead display this complex seizure threshold phenotype. However, human 15q13.3 deletion carriers mainly display absence seizures and some rat models of absence epilepsy are protected from clonic seizures.^7, 8, 19, 20^ Furthermore, the PTZ-induced seizure assay tests the excitability of neurocircuitry involved in seizures, but does not directly model epilepsy. We have not observed spontaneous convulsive seizures; however, it would be relevant in future studies to examine whether seizures develop in mice older than 6 months, and whether spontaneous absence seizures develop as previous EEG studies in hemizygous mice, indicating a lowered threshold to absence seizures.^9^ It is striking that seizure-related alterations are prominent phenotypes in both humans and mice with the deletion and that they show gene-dosage-dependent effects in both. Therefore, we think that these alterations are very likely to be related and that further exploration of the seizure phenotypes in the mice might provide an understanding of the epilepsy phenotypes in humans.

We also observed changes relevant to the three hallmarks of autism; (1) stereotypic repetitive behavior, (2) change in social behavior, and (3) communication abnormalities.^21^ The repetitive jumping that we observed in Df(h15q13)^−/−^ mice indicates that these mice mimic the restricted and repetitive behaviors seen in patients suffering from autism. Similarly, the decrease in ultrasonic vocalization that we observed in Df(h15q13)^−/−^ pups indicates that the abnormal communication observed in patients suffering from autism is mirrored in the Df(h15q13)^−/−^ mice.^22^ For both repetitive behavior and ultrasonic vocalization we observed changes in the same direction in Df(h15q13)^+/−^ mice that were not significant after correcting for multiple testing. A previous report of similar changes in another hemizygous deletion model further supports that there is an effect in the hemizygotes and thus a gene-dosage dependent effect on these phenotypes.^10^ In contrast, the increase in social sniffing that we observed in the three-chambered social approach assay for Df(h15q13)^−/−^ mice, is unexpected given the impairment of social behavior characteristic for patients with autism.^21^ There was a nonsignificant trend in the same direction in Df(h15q13)^+/−^ mice, but this is not supported by previous work in another hemizygous 15q13.3 deletion mouse model.^10^ The three-chambered social-approach assay quantifies how much time the mice use exploring a novel mouse, which can be interpreted as social interest, but it does not qualitatively assess social behavior.^23^ Thus, it indicates a social behavior abnormality of Df(h15q13)^−/−^ mice, but further studies are needed to describe this in detail. The social phenotype of the Df(h15q13)^−/−^ mice could be further investigated in reciprocal interaction, where Kogan *et al.*^10^ observed decreased social interaction for their hemizygous 15q13 mouse model.

Nest building has been proposed as a behavior with relevance to negative symptoms in schizophrenia.^24^ And we observed a gene-dosage-dependent nest-building impairment in the Df(h15q13)^−/−^ and Df(h15q13)^+/−^ mice.^17^ We also detected a decrease in acoustic startle response and prepulse inhibition in Df(h15q13)^−/−^ mice, whereas Df(h15q13)^+/−^ only had decreased acoustic startle response in accordance with previous characterization of the hemizygotes.^9^ Without direct tests of hearing, it cannot be ruled out that impaired hearing contribute to this phenotype in Df(h15q13)-/- mice. However, this finding further support the Df(h15q13)^−/−^ mice as a model for aspects of schizophrenia, as impairments in PPI have been reported in several psychiatric disorders and particularly in schizophrenia.^25^

Cognitive disabilities are another relevant group of phenotypes in relation to both schizophrenia and autism. As a measure of cognitive function we tested spatial learning and memory in the Morris water maze test, where Df(h15q13)^+/−^ mice were previously shown to have a deficit in long-term spatial memory.^9^ The Df(h15q13)^−/−^ mice were unable to learn the location of the hidden platform, and were unable to find the platform in the visual test where a flag is placed on top of the platform. Possible reasons for this might be; (1) cognitive impairments that prevent the mice from using the visual cues to guide them to the platform, (2) visual impairments that prevent the mice from seeing the visual cues and the flag, (3) repetitive movements or absence seizures and (4) a combination of these. In the present study, we observed normal visual placing response and corneal response in the Df(h15q13)^−/−^ mice, so the mice are not blind. However, the *Trpm1* gene in the 15q13.3 region has been linked to deficits in visual light response,^26, 27^ and mild visual impairment might prevent the Df(h15q13)^−/−^ mice from detecting the visual cues in the Morris water maze test. Further assessment of visual function in the Df(h15q13)^−/−^ mice is needed to elucidate this.

Owing to the difficult interpretation of the Morris water maze data, we performed a cognitive test that did not rely strongly on visual function—the fear-conditioning test. In the cue test the Df(h15q13)^−/−^ mice displayed a deficit in auditory cued fear memory (Figure 6d), indicating that memory is indeed impaired in Df(h15q13)^−/−^ mice, consistent with the high penetrance of intellectual disability in the 15q13.3 microdeletion syndrome.^2^

For most of the observed behavioral Df(h15q13)^−/−^ phenotypes there was a similar but less pronounced change in the Df(h15q13)^+/−^ mice (Table 2). Clear examples of such gene-dosage-dependent phenotypes were the reduced incidence of PTZ-induced clonic seizures, impaired nest building and reduced acoustic startle response. A few phenotypes such as PPI deficits were only present in the Df(h15q13)^−/−^ mice. Such phenotypes may be irrelevant to the hemizygous deletion, but considering the variable phenotype in the human hemizygous microdeletion syndrome, some may be dependent on interaction with environmental or additional genetic factors. Thus, it could be interesting to test whether hemizygous Df(h15q13)^+/−^ mice had, for example, PPI phenotypes if subjected to environmental challenge or bred in a different genetic background.

It is unknown which gene(s) in the 15q13.3 region that drives the phenotypes seen in humans and mice affected by the hemizygous and homozygous microdeletions. CHRNA7 has been the most popular candidate, as several cases of deletions encompassing only CHRNA7 have been described in patients with neuropsychiatric disorders.^3, 28, 29, 30^ The characterization of Df(h15q13)^−/−^ mice allows comparison with mice with single homozygous gene deletions. Single gene knockout mice have been described for three of the genes in the 15q13.3 region, *KLF13*, *TRPM1* and *CHRNA7*.

Klf13^−/−^ mice are reported as normal overall and do not have reduced weight like the Df(h15q13)^−/−^ mice.^31, 32^ Klf13^−/−^ mice characterization has focused on blood composition and no behavioral tests of the Klf13^−/−^ mice have been described. Trpm1^−/−^ mice display reduced visual sensitivity to light.^26, 27^ As Trpm1 is mainly expressed in the retina and essentially not in the brain, it is unlikely to be a major driver of the behavioral and physiological phenotypes of the Df(h15q13)^−/−^ mice. However, it might contribute to the impaired water-maze performance through effects on vision. Chrna7^−/−^ mice have been thoroughly studied, but have very subtle phenotypes.^33, 34, 35^ In contrast to Df(h15q13)^−/−^ mice, Chrna7^−/−^ mice develop normally, have unaltered seizure threshold, display normal acoustic startle response and normal prepulse inhibition, do not display deficits in memory assessed by the Morris water maze and auditory fear-conditioning tests.^35^ Consequently, it seems unlikely that Chrna7 alone accounts for the phenotypes that we observed in the Df(h15q13)^+/−^ and Df(h15q13)^−/−^ mice. Thus, one or more of the other genes in the region must be important for the phenotypes in Df(h15q13)^−/−^ mice and by inference in the human 15q13.3 deletion.

In conclusion, the behavioral phenotypes of the Df(h15q13)^−/−^ mice reported here demonstrate that Df(h15q13)^−/−^ mice are relevant as a model of both the hemizygous and the rare homozygous 15q13.3 microdeletion syndromes. The phenotypes related to epilepsy, autism and schizophrenia that we observed in the Df(h15q13)^−/−^ mice support that they can be used to explore how 15q13.3 deletion can lead to these disorders. Importantly, the Df(h15q13)^−/−^ mice might also be useful for studying the biological overlap between the disorders.

Mechanistic exploration is much simpler when phenotypes are strong, and the many gene-dosage-dependent phenotypes indicate that the Df(h15q13)^−/−^ model may be used to complement the Df(h15q13)^+/−^ model to explore the underlying biology of 15q13.3-associated disorders. Currently, the treatment options for epilepsy, autism and schizophrenia are not optimal and drug development efforts have been hampered by the lack of biological understanding of these disorders. If the Df(h15q13)^−/−^ mice can contribute to a better mechanistic understanding of biological changes underlying epilepsy, autism and schizophrenia they might ultimately facilitate drug discovery efforts in relation to these disorders.

## Figures and Tables

**Table 1:** Sample sizes for each experiment

	*WT*	*+/−*	*−/−*
Brain weight	12	—	9
Body weight	17	52	9
Grip strength	6	6	4
Locomotion	11	9	8
Early-stage seizure	7	6	5
Myoclonic jerks	7	6	5
Clonic seizure	7	6	5
Repetitive jumping	13	13	10
Three-chambered social approach	7	6	6
Ultrasonic vocalization	19	53	6
Nest building	8	10	6
Acoustic startle response	11	13	8
Prepulse inhibition	11	13	8
Morris water maze	6	6	4
Cue test (fear conditioning)	9	9	6

Abbreviation: WT, wildtype. +/-, Df(h15q13)+/- mice. -/-, Df(h15q13)-/- mice.

**Table 2:** Df(h15q13)^−/−^ fraction of all animals at weaning and survival into adulthood depends on weaning conditions

*Condition*	*At weaning*			*Adulthood*			
	*Total*	*−/−*	*−/− Fraction*	*Total*	*−/−*	*−/− Fraction*	*Binomial test*
Weaning at 3 weeks	162	19	0.117	147	6	0.041	*P*<0.001
Weaning at 4 weeks	101	12	0.119	98	11	0.112	*P>*0.05
Weaning at 4 weeks+supplements	403	50	0.124	399	50	0.125	*P>*0.05

At weaning: genotyping at weaning, 3 or 4 weeks after birth. Df(h15q13)^−/−^ fraction compared with birth ratio by binomial test (birth ratio was 0.20). Adulthood: 8 weeks. Total: total number of mice. −/−: number of Df(h15q13)^−/−^ mice. −/− fraction: Df(h15q13)^−/−^ fraction of mice compared with fraction at weaning by binomial test. Supplements: nutritional supplements were added to the breeding cages. Pairwise testing probability of survival into adulthood assuming binomial numbers and using an approximate Gaussian test: weaning at 3 weeks vs weaning at 4 weeks *P*<0.05, weaning at 3 weeks vs weaning at 4 weeks+supplements *P*<0.01, weaning at 4 weeks vs weaning at 4 weeks+supplements *P>*0.05.

**Table 3:** Behavioral phenotypes expressed qualitatively compared with WT, which is designated ‘0’

	*WT*	*+/-*	*-/-*
Early-stage seizure	0	+	++
Myoclonic jerks	0	+*	0
Clonic seizure	0	−*	−−
Repetitive jumping	0	(+)	++
Social preference	0	(+)	++
Ultrasonic vocalization	0	(−)*	−−
Nest building	0	−	−−
Acoustic startle response	0	−	−−
Prepulse inhibition	0	(−)	−−
Cue test (fear conditioning)	0	(−)	−−

Abbreviation: WT, wildtype.

Decrease or increase in the Df(h15q13)+/− compared with WT, is indicated as ‘−’ or ‘+’, respectively. Changes that are not statistically significant are indicated in brackets. Stronger decrease or increase in the Df(h15q13)−/− than Df(h15q13)+/− is indicated as ‘—’ or ‘++’, respectively. ‘*’ previous reports show a significant change in the same direction for Df(h15q13)+/− mice compared with WT.

^9, 10^
