# Supplementary material for: 15q13.3 homozygous knockout mouse model display epilepsy-, autism- and schizophrenia-related phenotypes
Source: Transl Psychiatry. 2016 Jul 26;6(7):e860–. doi: 10.1038/tp.2016.125 (PMC5545711; doi:10.1038/tp.2016.125)
Supplement: Supplementary Figures [file tp2016125x1.doc]

# Legends to supplementary figures

**Figure S1. Brain weight of Df(h15q13)^-/-^ mice.** n=9-12 for each genotype. Unpaired two-sided t-test indicated. Data presented as Mean±SEM. *p< .05; **p< .01; ***p< .001.

**Figure S2. Abnormal seizure response to PTZ in Df(h15q13)^-/-^ mice.** Mice received PTZ 40 mg/kg subcutaneously and were observed for 30 min and scored for seizures as described in materials and methods. **A-C│**Batch A females. **D-F│**Batch B females. **A│**Time in early stage seizure. n=2-4. Kruskal-Wallis test, NS. **B│**Number of myoclonic jerks during a 30 min period after PTZ administration. n=5-8. One-way ANOVA, NS. **C│**Incidence of clonic seizures. n=5-8. Logrank test, p<0.05. WT vs Df(h15q13)^+/-^ NS, WT vs Df(h15q13)^-/-^ p<0.05, Df(h15q13)^+/-^ vs Df(h15q13)^-/-^ NS. **D│**Time in early stage seizure. n=10-13. One-way ANOVA, P<0.05. Tukey’s multiple comparisons indicated. **E│**Number of myoclonic jerks during a 30 min period after PTZ administration. n=10-13. One-way ANOVA, P<0.05. Tukey’s multiple comparisons indicated. **F│**Incidence of clonic seizures, no Df(h15q13)^-/-^ mice had clonic seizures. n=10-13. Logrank test, p<0.001. WT vs Df(h15q13)^+/-^ P<0.05, WT vs Df(h15q13)^-/-^ p<0.001, Df(h15q13)^+/-^ vs Df(h15q13)^-/-^ P<0.05. Data (**A, B, D & E**) presented as Mean±SEM. *p< .05; **p< .01; ***p< .001.

**Figure S3. Early stage seizure first eight minutes.** Mice received PTZ 40 mg/kg subcutaneously and were observed for 30 min and were scored for seizures as described in materials and methods. n=5-7. Time in early stage seizure during the first eight minutes (i.e. before any mice had clonic seizures). One-way ANOVA, NS. Data presented as Mean±SEM. *p< .05; **p< .01; ***p< .001.

**Figure S4. Repetitive jumping/crawling.** Mice were placed in individual cages and observed for ten minutes. Quantification of stereotypic jumping or crawling in the corner of the cage is shown here. **A│**Batch A females, n=3-5. Kruskal-Wallis test, NS. **B│**Batch A males, n=8-12. Kruskal-Wallis test, P<0.05. Dunn’s multiple comparisons indicated . **C│**Batch B males, n=8-12. One-way ANOVA, NS. Data presented as Mean±SEM. *p< .05; **p< .01; ***p< .001.

**Figure S5. Progressive** **startle. A│**Batch A females. n=3-6. Two-way ANOVA, no significant interaction between intensity and genotype. Main effect of pulse intensity (P<0.01), but not of genotype. **B│**Batch B females. n=10-13. Two-way ANOVA, no significant interaction between intensity and genotype. Main effects of pulse intensity (P<0.0001) and genotype (P<0.0001). Tukey’s multiple comparisons indicated. **C│**Batch B males. n=4-8. Two-way ANOVA, no significant interaction between intensity and genotype. No main effect of pulse intensity, but of genotype (P<0.0001). Tukey’s multiple comparisons indicated. Data presented as Mean±SEM. *p< .05; **p< .01; ***p< .001.

**Figure S6. Prepulse inhibition at varying prepulse intensities. A│**Batch A females, n=3-6. Two-way ANOVA, no significant interaction between prepulse intensity and genotype, no main effect of prepulse intensity, but of genotype (P<0.001). Tukey’s multiple comparisons of genotype groups indicated. **B│**Batch B females, n=10-13. Two-way ANOVA, no significant interaction between prepulse intensity and genotype, main effect of prepulse intensity (P<0.0001) and of genotype (P<0.0001). Tukey’s multiple comparisons of genotype groups indicated. **C│**Batch B males, n=4-8. Two-way ANOVA, no significant interaction between prepulse intensity and genotype. Main effect of prepulse intensity (P<0.001) and of genotype (P<0.001). Tukey’s multiple comparisons of genotype groups indicated. Data presented as Mean±SEM. *p< .05; **p< .01; ***p< .001.
