# Supplementary figures and images for: 15q13.3 homozygous knockout mouse model display epilepsy-, autism- and schizophrenia-related phenotypes
Source: Transl Psychiatry. 2016 Jul 26;6(7):e860–. doi: 10.1038/tp.2016.125 (PMC5545711; doi:10.1038/tp.2016.125)

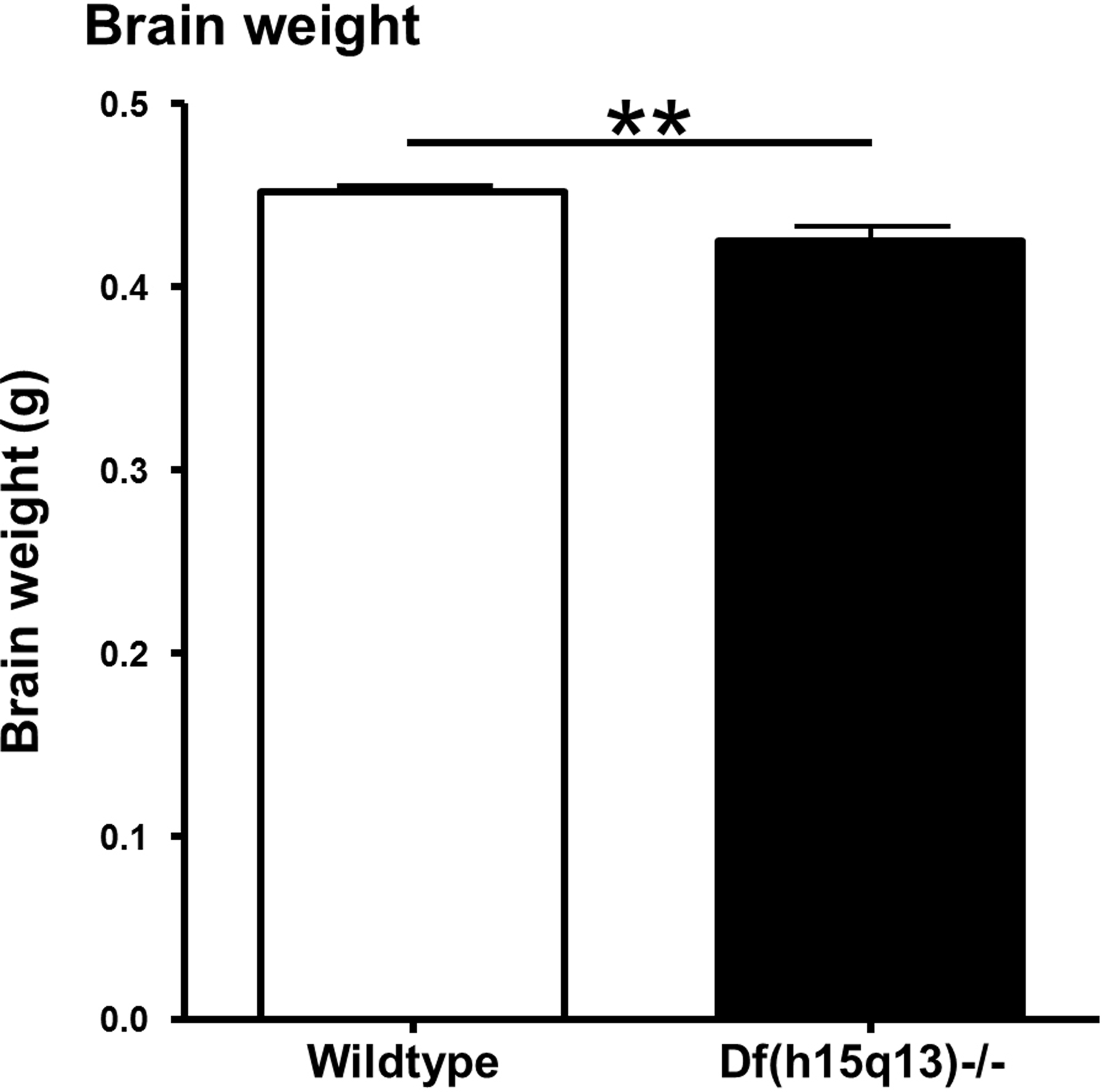

Supplement: Supplementary Figure 1 [file tp2016125x2.tif]

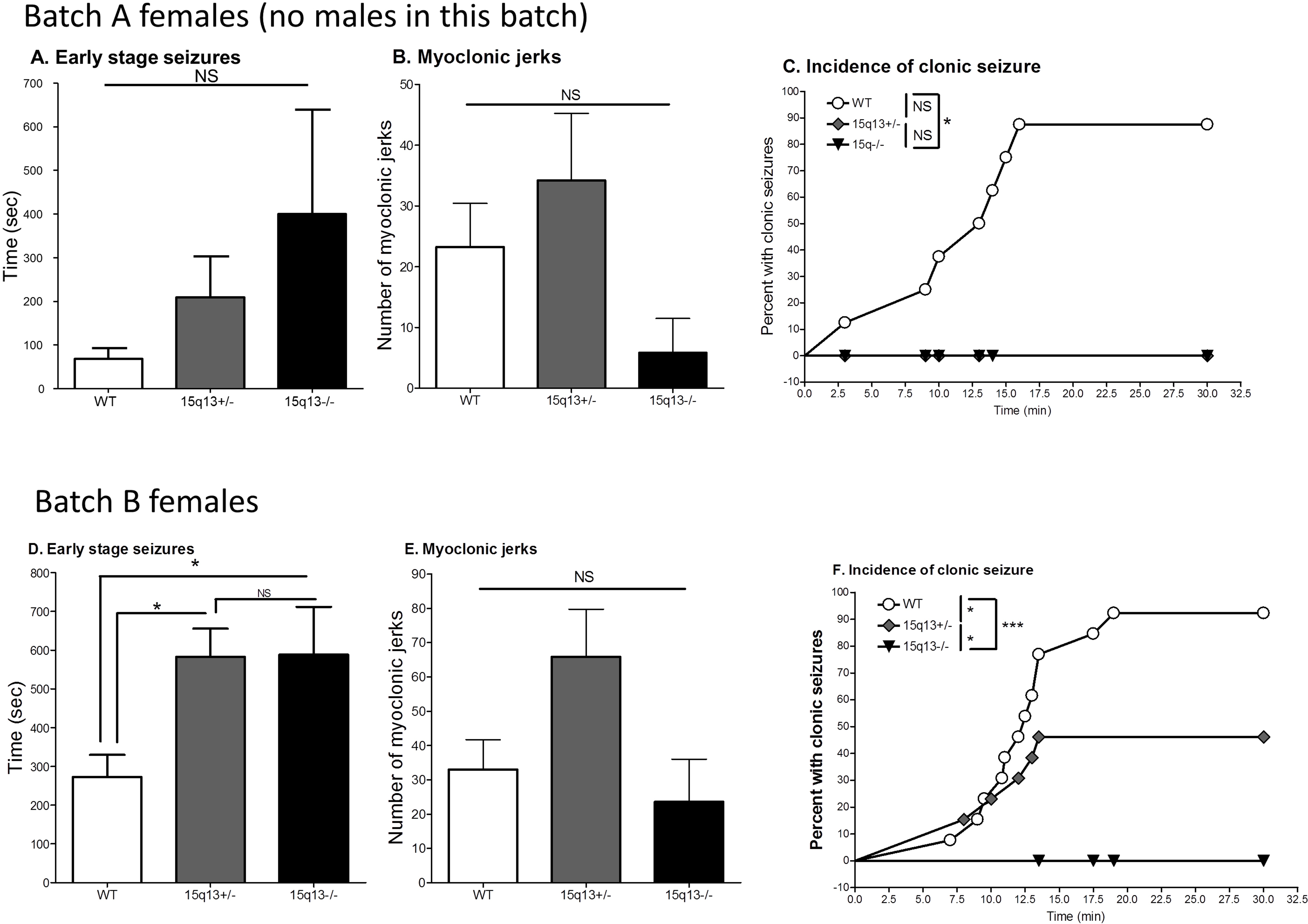

Supplement: Supplementary Figure 2 [file tp2016125x3.tif]

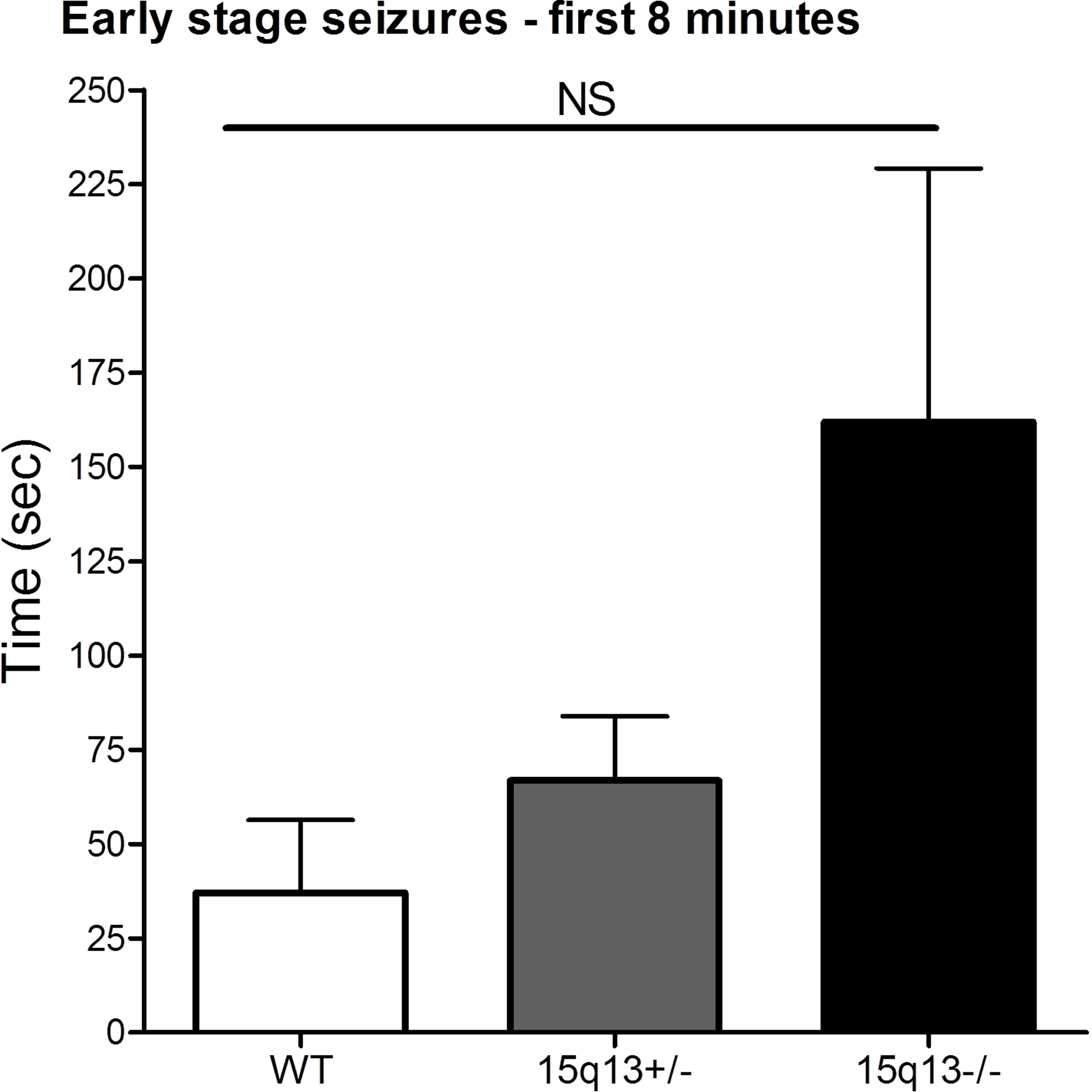

Supplement: Supplementary Figure 3 [file tp2016125x4.tif]

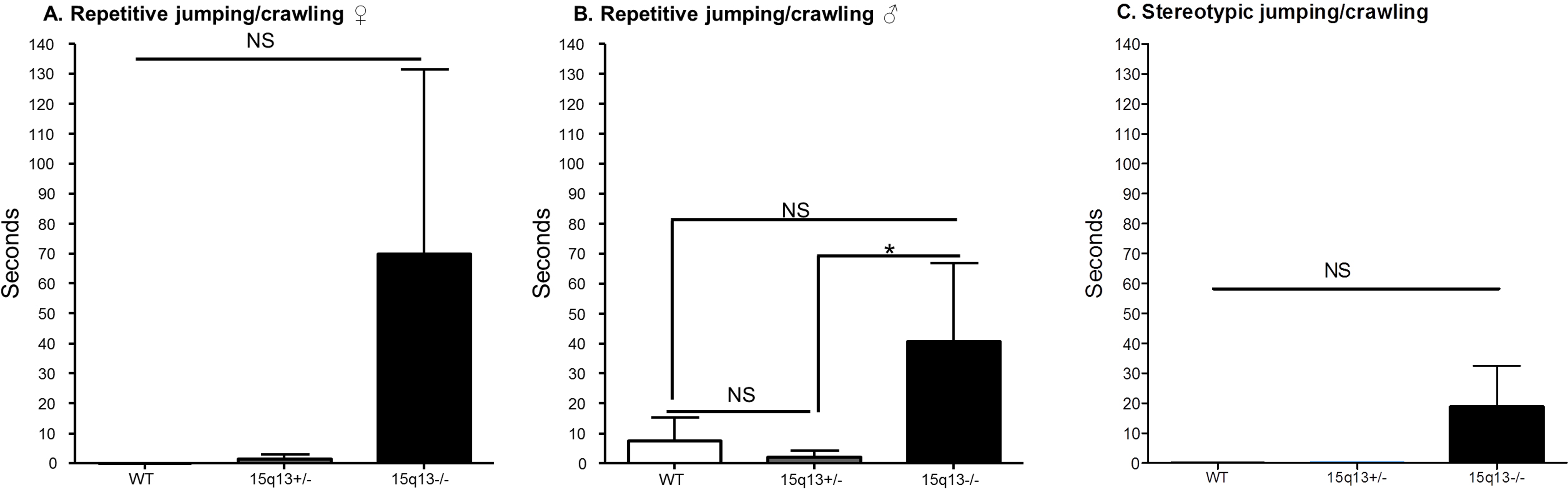

Supplement: Supplementary Figure 4 [file tp2016125x5.tif]

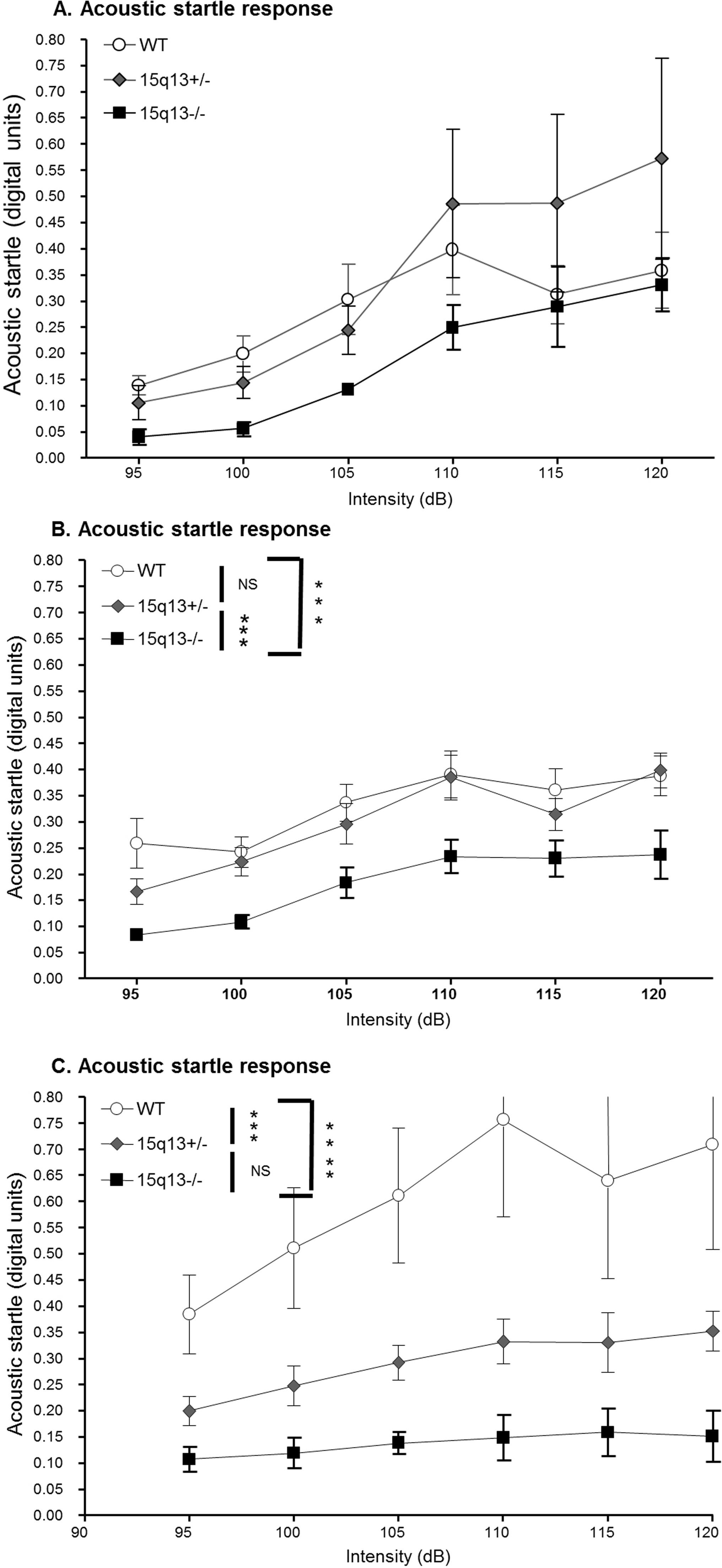

Supplement: Supplementary Figure 5 [file tp2016125x6.tif]

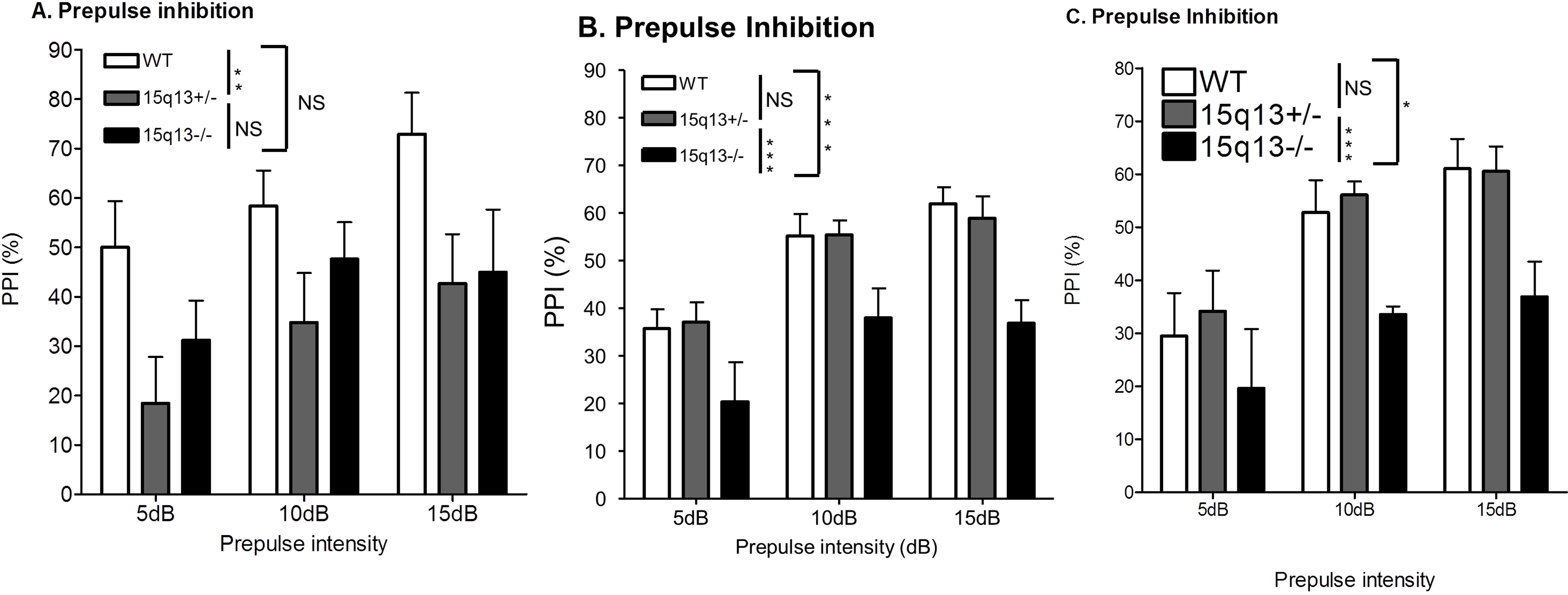

Supplement: Supplementary Figure 6 [file tp2016125x7.tif]
